# Strong biomechanical constraints on young children's mental imagery of hands

**DOI:** 10.1098/rsos.140118

**Published:** 2014-12-10

**Authors:** Kaoru Sekiyama, Toshiro Kinoshita, Takahiro Soshi

**Affiliations:** 1 Division of Cognitive Psychology, Kumamoto University, Kumamoto, Japan; 2 School of Systems Information Science, Future University, Hakodate, Japan

**Keywords:** body schema, motor imagery, mental rotation, hand, child, development

## Abstract

Mental rotation (MR) of body parts is a useful paradigm to investigate how people manipulate mental imagery related to body schema. It has been documented that adult participants use ‘motor imagery’ for MR of hands: a behavioural indication is a biomechanical effect, that is, hand pictures in orientations to which imitative hand movement would be biomechanically difficult require longer response times to be visually identified as the left or right hand. However, little is known about the typical developmental trajectory of the biomechanical effect, which could offer clues to understanding how children acquire the ability to manipulate body schema. This study investigated developmental changes in the biomechanical effect in schoolchildren. Eighty-four children (from 6 to 11 years old, grouped into 1st, 2nd, 3rd, 4th and 5th graders) and fifteen adults made hand laterality judgements in an MR paradigm. The results indicated that the biomechanical effect is stronger for younger children, and that there is a transitional period (around 7–8 years) during which children shift from action execution to imagery in manipulating body schema. The results suggest that mental imagery of hands has a stronger motor aspect in the transitional period than later in childhood and adulthood.

## 2. Introduction

The concept of ‘body schema’ comes from a postural model in the brain to account for the inability to appreciate postural changes following cerebral lesions [1]. Body schema is now thought to be fundamental for bodily awareness, as well as motor imagery [2,3], and can thus form a basis for action–perception links and embodied cognition, for example, activation of leg-related brain areas in recognizing leg-related action words [4–6]. Although the earlier concept was originally focused on a proprioceptive dimension, recent evidence indicates that body schema represent not only proprioceptive information, but also relations among different modalities concerning body parts, including proprioceptive, visual and motor information [7–10]. In our working definition, the simultaneous nature of multiple sensory and motor signals from body parts enhances connections among brain regions, generating a stable network representing a body schema (like ‘cell assemblies’ [11]). One clear demonstration of the multimodal nature of body schema is motor imagery generated for visually presented body parts, as described below.

Despite the recent advancement of our understanding of adult body schema, the developmental trajectory of body schema in childhood has been less explored. To elucidate how children acquire the ability to manipulate body schema, we investigated developmental changes in mental imagery based on body schema. Although the term ‘motor imagery’ is often used for this kind of imagery [12], ‘visuomotor imagery’ may be more accurate to describe the multimodal aspects of bodily imagery [4]. Mental rotation (MR) of hands, described below, is a useful paradigm to investigate body schema and visuomotor imagery.

In the hand MR task, pictures of human hands in various orientations are presented, and participants are asked to decide whether a picture shows a left or right hand without moving their own hands [13,14]. If participants can perform the task accurately, the inference is that they have an appropriate mental representation for hands. Response time (RT) data indicate that hands presented in an upright orientation (fingers up) or close to this orientation are identified most quickly; thus, these orientations are thought to be canonical based on the body schema for hands [13–15].

**Figure 1.**
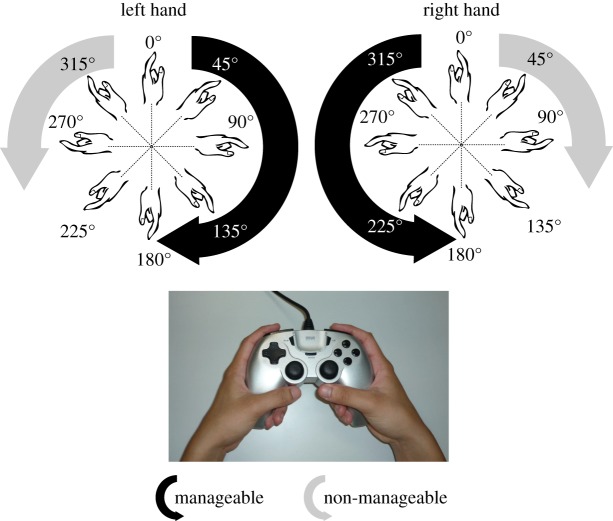
Rotation angles in the picture plane for MR with the eight orientations by picture-plane rotation (defined by the clockwise rotation from upright with a 45^°^ step). The manageable direction was clockwise (counter-clockwise) for the left (right) hand.

More importantly, RTs typically indicate that participants solve this task by mentally simulating movements of their own hands to bring the imagined hand towards the stimulus orientation. A behavioural measure of such action simulation is the pattern of RTs indicating ‘biomechanical constraints’: greater RTs result for those orientations that the arm and hand cannot easily reach with a real movement because of biomechanical constraints of arm joints [13,14,16,17]. More specifically, outward rotation is generally non-manageable, while inward rotation is more manageable, in the picture plane (figure 1). This was confirmed by comparing RTs for the hand MR task and subjective ratings for biomechanical ‘manageability’ in imitative hand movements [16,17]. The RT pattern indicating biomechanical constraints is now thought to represent ‘motor imagery’. Although MR of body parts is not the only experimental paradigm used to study motor imagery [18,19], the biomechanical influence on RTs is a useful measure. Furthermore, a merit of the hand MR task is the existence of correct responses (a ‘left’ response is correct for a left-hand stimulus), which provides objective validity for imagery-related RTs.

Neuroimaging studies have revealed that the hand MR task constantly activates the dorsal premotor cortex [12,20–24], whereas non-body MR tasks do not [21,23]. More precisely, the activation of the dorsal premotor area was often localized along the precentral sulcus [12,20–22]. Therefore, the posterior part of the dorsal premotor cortex may be a sign that the hand MR task involves motor planning or visuomotor imagery. Some studies also implicated the primary motor/somatosensory area for the hand MR task [23,25].

In human brain development, speed of maturation varies considerably among different cortical areas [26]. The dorsal premotor area, a critical region for visuomotor imagery, is one of the late-matured areas, and maturation is completed only in adolescence. This is in sharp contrast to early maturation in the primary areas such as the primary somatosensory and motor areas, which mature by 5 years of age. Considering the slow development of the dorsal premotor area, young children's manipulation of body schema may be immature and they may respond to the hand MR task in a way quite different from that of adults. However, the hand MR task has been less used to understand typical development of motor imagery or body schema in childhood, although it has been widely applied to study motor imagery deficits, including developing populations (e.g. [27–29]).

Combining the neuroimaging findings on adult brain activation for the hand MR task [12,20–25] and those on differential maturation of various brain regions [26], we propose a ‘primary area hypothesis’. This hypothesizes that young children have difficulty in the hand MR task mostly due to the immaturity of the dorsal premotor cortex, and their internal processes for the hand MR task would involve the early matured primary motor and somatosensory areas more heavily than those of adults. This expectation predicts that young children's manipulation of body schema should rely more heavily on information related to action execution and therefore exhibit stronger biomechanical constraints on RTs for the hand MR task, because these primary areas are more directly associated with somatomotor (thus biomechanical) processing compared with the visuomotor associating nature of the premotor area [30]. Although this is a speculative view, it seems to be in accordance with what earlier developmental theorists argued, that is, young children's knowledge representation depends on action execution before they develop adult-like iconic or visual representation [31,32].

In fact, our prediction is consistent with a previous finding that children around 6 years old were more affected by current proprioceptive information (posture of their own hands) than adults in the hand MR task [33]. However, while few studies have examined biomechanical constraints on young children's motor imagery [33,34], a previous study reported inconsistent results with our prediction, that is, biomechanical constraints were weaker for 5-year-olds than for 7-year-olds and adults in the hand MR task [35]. To solve these inconsistencies, we examined developmental trajectory of biomechanical effects in optimal experimental settings to evoke motor imagery.

Krüger & Krist [35] devised a hand MR task in a matching-to-sample paradigm to study young children including 5-year-olds. Although having a merit of being child-friendly, the simultaneous presentation of multiple hands in the matching-to-sample paradigm may have induced visual comparison processes rather than accessing body schema in some age groups [36]. Visual MR comparing two familiar objects is relatively easy and can be performed by 4- or 5-year-old children [37–39], while comparing two complex stimuli such as the well-known Shepard-type cube figures is very difficult even for 8-year-olds [40]. Our focus was on how participants generate an image of their own hands based on body schema.

We used a standard hand MR task in which rotated hand pictures are presented one at a time so that each one is compared with each participant's body schema. To make sure that motor imagery was evoked, depth variation was introduced by presenting the back, thumb-side and palm of each hand [41]. If young children primarily recruit the early matured primary motor and somatosensory areas in motor imagery while adults mainly recruit the dorsal premotor area, greater biomechanical constraints on RTs are expected in younger children. We tested this prediction in schoolchildren (ranging in age from 6 to 11 years) and adults, and attempted to delineate the developmental trajectory of body-related imagery. The lower age limit was set for the feasibility of the task based on our pilot study. The age range was to capture the rapid development of motor imagery during childhood [34], for which most cortical regions seem to reach their peak thickness by around age 10–11 years, although further neurodevelopmental refinement follows until adulthood [42].

## 3. Material and methods

### 3.1 Participants

Table 1 summarizes the demographic properties of the participants (*n*=99). Japanese children (range from 6.4 to 11.4 years old; *n*=84) were recruited from a regional primary school and children's after-school home. Japanese undergraduate students (mean 19.62 years old; range from 18.7 to 21.2 years old; *n*=15) were recruited as an adult group. The grouping of children was based on school grade, but age was used for labelling each group (6 years for the 1st grade, 7 years for the 2nd grade and so on) for comprehensiveness (but also see the electronic supplementary material for data analyses with grouping by age). The groups did not differ significantly in terms of sex ratios (*χ*^2^-test: $\chi_{\left(5\right)}^{2}=2.163$, *p*=0.826). All participants were right-handed and had normal or corrected-to-normal vision. Handedness was checked by a laterality questionnaire simplified from the Edinburgh Handedness Inventory (only five items were used, with a criterion of four out of five) for younger children.

**Table 1.** Summary of participant demographic characteristics (*n*=99). Age groups were defined by school grade, but labelled with age.

age group	*n*	male/female	mean age	school grade
6-year-olds	20	11/9	6.82	1st
7-year-olds	18	11/7	7.96	2nd
8-year-olds	16	11/5	8.80	3rd
9-year-olds	15	9/6	9.84	4th
10-year-olds	15	11/4	10.86	5th
19-year-olds	15	11/4	19.62	UG

### 3.2 Materials

**Figure 2.**
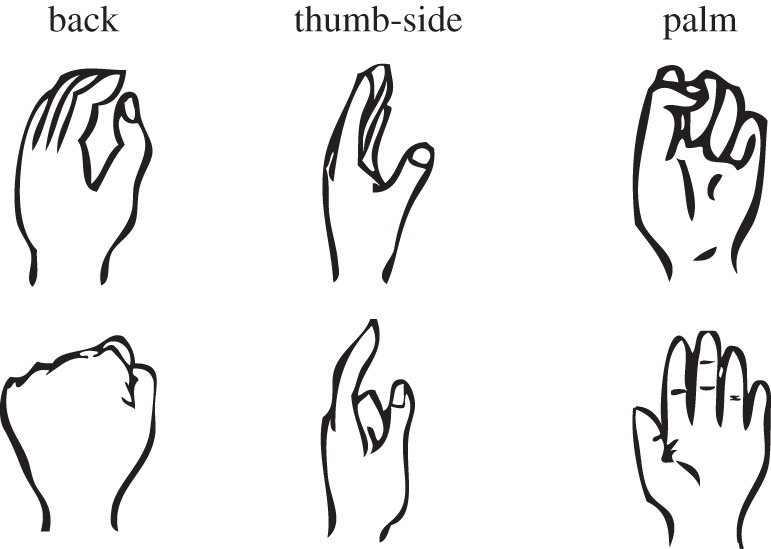
Hand drawings used in test (upper) and practice (lower) trials. The six types consisted of three degrees of depth rotation (back, thumb-side and palm). The upper three drawings and their mirror images were presented in one of eight orientations (figure 1) in test trials.

Stimuli were the hand drawings in figure 2 (left hands) and their mirror images (right hands). In test trials, they were presented in one of eight orientations (figure 1). The eight angles were defined by clockwise rotation in 45^°^ steps from 0^°^ (upright) to 315^°^ in the picture plane. Based on the internal process with biomechanical constraints, anticipated peak angles of RT were 225^°^ (and 270^°^) for the left hand and 135^°^ (and 90^°^) for the right hand [14]. The longest axis of the stimulus subtended to approximately 8^°^ of the visual angle.

In the test trials, three types of hands were used (upper three images in figure 2). Prior to the test trials, there were step-by-step practice blocks, and stimuli not used in the test trials were mainly used in the practice blocks (lower three images in figure 2).

### 3.3 Procedure

Throughout the practice and test trials, the procedure was basically identical. Participants were individually tested, seated at about 50 *cm* from the display of a laptop computer and holding a game controller with both hands with the thumb-side up (bottom of figure 1). The hands and game controller were placed under a desk so that they were not visible. A certain number of stimuli were presented, one at a time, in a random order. The participants were instructed to identify each hand as a left or right hand as accurately and quickly as possible without moving or seeing their own hands. Each stimulus was presented on the centre of a 15-inch display with a white background. A fixation point first appeared on the centre of the display for 1500 *ms*. Immediately after its disappearance, a hand stimulus was presented until a response was made. For responses, participants were instructed to press the right (left) button for the right (left) hand judgement with the right (left) index finger. Response correctness and RT were recorded in each trial with in-house software.

Based on a pilot experiment in which younger children showed considerable difficulty in the standard hand MR task, we introduced step-by-step practice blocks in this experiment. These practices included (1) left–right laterality judgements on upright hands with feedback about correctness, (2) laterality judgements on upright hands without feedback, (3) laterality judgements on rotated hands with feedback, (4) laterality judgements on rotated hands with the participant's hands visible and one of the participant's hands to be moved to imitate the stimulus, and (5) the same setting as the test trials. To observe a generalization effect of practice, stimuli not used in the test trials were used in most practice blocks.

In the test blocks, stimuli were the upper three images in figure 2, and eight orientations were used. The participants were presented the 48 hand drawings (left/right hands × 3 types × 8 angles), randomly, one at a time. No response feedback was provided. First, the participants were given about 20 trials with exactly the same procedure as the test (Practice 5). After that, 96 test trials were conducted in two blocks. The participants were asked to perform the task without moving or seeing their own hands. However, if younger children preferred moving their own hands, no further inhibition was used. During the test block, the experimenter observed the participants from behind and checked whether or not they moved their own hands during the task.

### 3.4 Data analyses

We first counted the number of participants who performed the task significantly above chance-level accuracy without moving their own hands (‘pass’) in each age group. For the 50% chance level, 64.58% (62/96) was the criterion used to determine whether a correct response rate was significantly above chance ($\chi_{\left(1\right)}^{1}=4.17$, *p*<0.05). Participants who performed the task above chance, but moved their own hands (‘move’) and those who performed below chance (‘fail’) were also categorized into different groups. Distributions of participants were compared overall between groups with a *χ*^2^-test (3 response categories × 6 groups). Follow-up pair-wise group comparisons were made using *χ*^2^-tests (3 response categories × 2 groups).

The analysis of RTs (*n*=67) excluded those who failed and moved their own hands during the task (all of the 6-year-olds, three of the 7-year-olds and two of the 8-year-olds). In addition, in the ‘pass’ group, those who had one or more angles in either of the hands for which the error rate was higher than 83% (only one correct response out of six repetitions) were also excluded (five of the 7-year-olds and two of the 8-year-olds) to obtain a mean RT at each angle.

To examine the influence of biomechanical constraints on the hand MR task for each participant group, error rates as well as RTs were compared between manageable angles (left hand: 45^°^–135^°^; right hand: 225^°^–315^°^) and non-manageable angles (left hand: 225^°^–315^°^; right hand: 45^°^–135^°^). These angles were determined based on a previous study [17]. For error rate analyses (*n*=99), a *χ*^2^-test (with Yates's correction for continuity) was used because data broke the normal distribution (Kolmogorov–Smirnov test for each group in each manageable and non-manageable conditions; significant deviations from normality were found in many cases). Individual correct and incorrect responses were counted for manageable and non-manageable angles across both hands and hand types for the *χ*^2^-test within each group. In addition, to examine group difference effects on the influence of manageability, the manageable/non-manageable ratio of (1 – error rate) was calculated for each individual, after logarithmical transformation of per cent correct score. Group differences in this ratio were examined using the Kruskal–Wallis test. Depending on the output, paired comparisons between groups were conducted with the Mann–Whitney test.

RTs (*n*=67) for correct responses were compared between manageable and non-manageable angles by a repeated measures ANOVA (manageability × laterality × group) with the within-subject variables of manageability and laterality. Individual mean RTs were calculated for manageable and non-manageable angles for each hand across hand types. Additionally, the internal consistency of RT–angle functions for children within each grade was tested by Cronbach's *α* coefficient, based on the reliability criterion of *α*>0.7.

To investigate developmental changes in biomechanical constraints on the RT–angle function, we examined pattern differences in the RT–angle relationship after removing absolute RT differences across ages. To do so, each of the individual mean RTs for 16 conditions was transformed to a *Z*-score by using each participant's grand mean and standard deviation of all 16 conditions (eight angles for each hand). By using means of normalized RTs for manageable (left hand: 45^°^–135^°^; right hand: 225^°^–315^°^) and non-manageable angles, an ANOVA (manageability × laterality × group) with repeated measures on manageability and laterality was performed. The differences between the normalized (*Z*-scored) mean RTs for manageable angles and for non-manageable angles were also examined by one-way ANOVA (group).

## 4. Results

### 4.1 Task execution of mental rotation by children and adults

**Figure 3.**
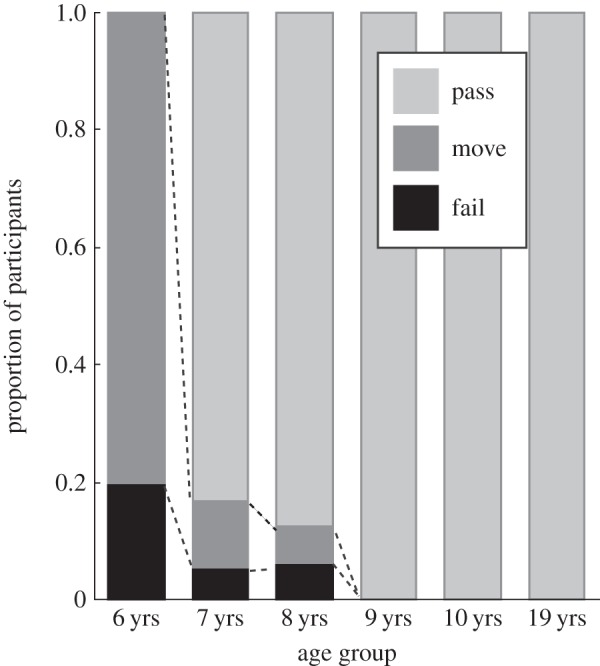
Proportion of participants categorized by performance level. ‘Pass’ indicates participants who performed with above chance accuracy without moving their own hands. ‘Move’ indicates those who performed above chance but moved their own hands. ‘Fail’ indicates those who performed below chance. The 6-year-old (1st-grade) children performed differently from any other age group.

All of the 6-year-olds (*n*=20) actually moved their own hands during the task to some extent (figure 3), especially for unfamiliar orientations. For such orientations, they often moved one hand at a time under the desk while holding the game controller with the other hand. Additionally, four 6-year-olds failed (below chance-level accuracy). Among the 7-year-olds, two moved hands during the task, and one failed. Among the 8-year-olds, one moved hands and one failed. The older children and the adults accurately completed the task without hand movements.

Task execution (pass, move, fail) differed significantly across ages based on *χ*^2^-analysis using the factors of task execution (pass, move, fail) and group (1st grade to adult) ($\chi_{\left(10\right)}^{2}=77.640$, *p*<0.0001, Cramer's *V* =0.63) (figure 3). Subsequent pair-wise comparisons showed that the 6-year-olds performed differently from any other age group ($\chi_{\left(2\right)}^{2}=27.660,V=0.85$; $\chi_{\left(2\right)}^{2}=28.948,V=0.90$; $\chi_{\left(2\right)}^{2}=35.000,V=1.0$; $\chi_{\left(2\right)}^{2}=35.000,V=1.0$; $\chi_{\left(2\right)}^{2}=35.000,V=1.0$, respectively versus 7-, 8-, 9- and 10-year-olds and adults; all *p*-values <0.0001). No other age differences were found in any other pairs. The results indicate that the standard hand MR task without moving hands is difficult for 6-year-old children.

### 4.2 Response times and error rates as a function of rotation angle

**Figure 4.**
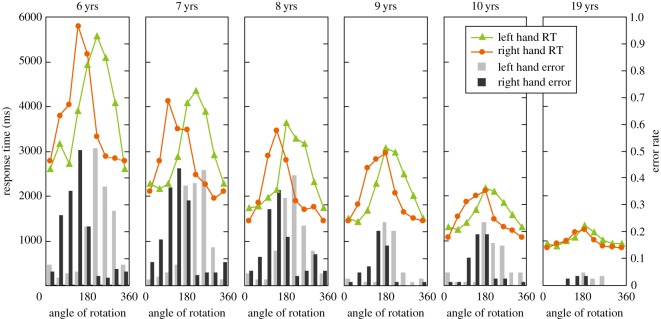
Mean RTs and error rates as a function of rotation angle in the picture plane. Mean RT curves for younger children (7- and 8-year-olds) showed a larger discrepancy between the left and right hands, including differences in peak angles for the maximum RTs. Error rates are based on all participants (*n*=99), and RTs are based on correct responses from those in the ‘pass’ group who produced reliable data for each angle (*n*=67, see Data analyses). The 6-year-olds either moved their own hands or failed (neither was a ‘pass’), and the data for reliable participants in the ‘move’ group (*n*=14) are shown only as a reference.

For each left and right hand, mean RTs and error rates are shown in figure 4 as a function of rotation angle. Group differences in RT–angle functions indicated developmentally weakened biomechanical constraints on hand MR. For example, the 7- and 8-year-olds showed fairly asymmetric RT curves for both hands. These children had peaks at 90^°^ or 135^°^ for the right hand, which correspond to the orientations with the greatest awkwardness of imitative hand movements [17], indicating that the 7- and 8-year-old children were strongly constrained by biomechanical manageability. By contrast, the peak angle in the older groups for the right hand was 180^°^ , although they still showed asymmetric RT curves.

The inter-individual consistencies in RT patterns were relatively high in each group (Cronbach's *α* coefficients for the left and right hands, respectively: 7 years, *α*=0.88 and 0.94; 8 years, *α*=0.87 and 0.91; 9 years: *α*=0.91 and 0.92; 10 years: *α*=0.89 and 0.92; 19 years: *α*=0.93 and 0.92), supporting the homogeneity of RT patterns within groups.

Error rates showed similar patterns to RTs, indicating the effects of biomechanical constraints (for example, the right hand showed more errors at 90^°^ and 135^°^ than in their mirror-reversed angles).

Statistical analyses for the group differences are presented below.

### 4.3 Biomechanical constraints on hand mental rotation in children

To examine the influence of biomechanical constraints on the hand MR task, error rates as well as RTs were compared between manageable (left hand: 45^°^–135^°^; right hand: 225^°^–315^°^) and non-manageable angles (left hand: 225^°^–315^°^; right hand: 45^°^–135^°^, as shown in figure 1). These angles were determined based on a previous study [17].

**Figure 5.**
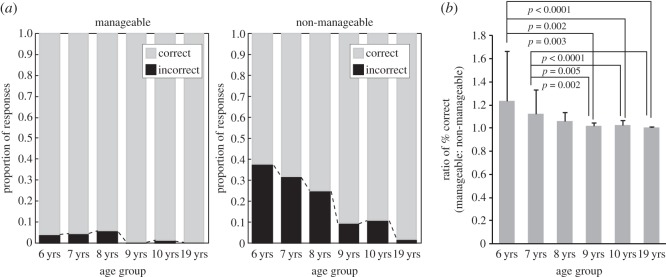
Proportion of frequencies of correct and incorrect responses. Manageable angles for the left hand were 45^°^, 90^°^ and 135^°^ (those for the right hand were 315^°^, 270^°^ and 225^°^), and non-manageable angles were mirror-reversed. (*a*) In each age group, more errors were made for non-manageable angles than manageable angles. (*b*) Group differences in the strength of biomechanical constraints are shown by the manageable:non-manageable ratio of (1−error rate).

Error rates were examined with a *χ*^2^-test in each group, using frequencies of correct and incorrect responses for manageable and non-manageable angles (figure 5*a*). In all groups, more errors were made for non-manageable than manageable angles (6 years: $\chi_{\left(1\right)}^{2}=247.195$, *p*<0.0001, *φ*=0.42; 7 years: $\chi_{\left(1\right)}^{2}=158.658$, *p*<0.0001, *φ*=0.35; 8 years: $\chi_{\left(1\right)}^{2}=76.075$, *p*<0.0001, *φ*=0.26; 9 years: $\chi_{\left(1\right)}^{2}=43.060$, *p*<0.0001, *φ*=0.20; 10 years: $\chi_{\left(1\right)}^{2}=40.925$, *p*<0.0001, *φ*=0.20; 19 years: $\chi_{\left(1\right)}^{2}=8.176$, *p*<0.005, *φ*=0.10). In a further analysis, group differences in the influence of manageability were examined. For this assessment, the manageable:non-manageable ratio of per cent correct (1−error rate) was calculated for each individual. If this ratio equals 1, errors are distributed symmetrically around 180^°^; larger ratios indicate larger error rates for non-manageable angles. A Kruskal–Wallis test found a significant main effect of group ($\chi_{\left(5\right)}^{2}=29.281$, *p*<0.0001). Subsequently, paired comparisons (Mann–Whitney tests) revealed that the 6-year-olds had a larger ratio compared with the 9-, 10- and 19-year-olds (6- versus 9-, 10- and 19-year-olds: *U*<62, *p*<0.003) (figure 5*b*); 7-year-olds had a larger ratio compared with 9-, 10- and 19-year-olds (7- versus 9-, 10- and 19-year-olds: *U*<59, *p*<0.005). Eight- and 9-, but not 10-year-olds also had larger ratios than adults (8- versus 19-year-olds: *U*=61.5, *p*=0.019; 9- versus 19-year-olds: *U*=59.5, *p*=0.026). These results indicated that overall, younger children experienced greater difficulty for non-manageable compared with manageable angles.

**Figure 6.**
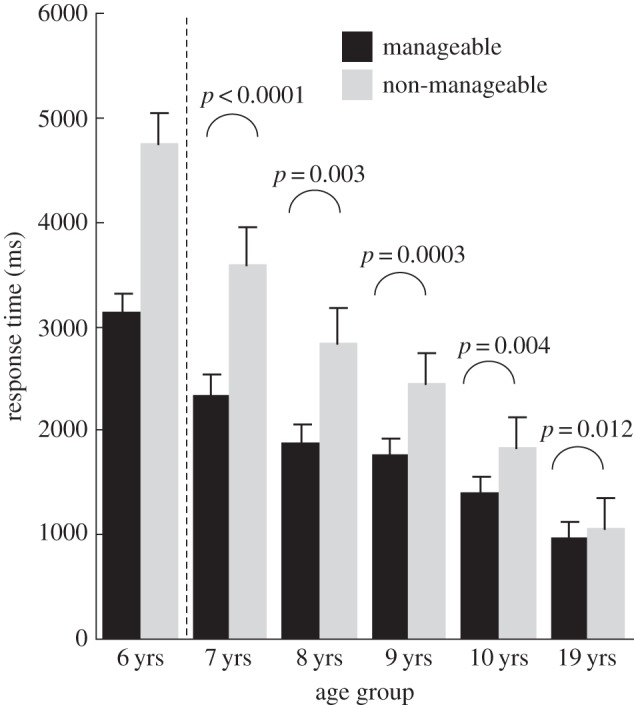
Mean RTs for manageable angles and non-manageable angles in each age group extracted from figure 4. The mean RT was longer for non-manageable angles than for manageable angles. The 6-year-olds were not included in the statistical analysis of RTs (*n*=67). Error bars are standard errors of means.

Biomechanical constraints also affected RTs (figure 6). Repeated measures ANOVA of manageability × laterality × group (2×2×5) showed significant main effects of manageability (*F*_1,62_=98.362, *p*<0.0001, *η*^2^=0.11), laterality (*F*_1,62_=7.441, *p*<0.01, *η*^2^=0.003) and group (*F*_4,62_=18.514, *p*<0.0001, *η*^2^=0.41), as well as an interaction of manageability × group (*F*_4,62_=8.259, *p*<0.0001, *η*^2^=0.04). The main effect of manageability indicated that both children and adults identified hands in manageable angles faster than those in non-manageable angles. A follow-up *t*-test in each group showed that this was also true for each age group (figure 6). As the significant interaction of manageability × group indicates, the biomechanical constraints tended to be more salient in younger children.

Overall, in all age groups, both error rates and RTs indicated biomechanical constraints measured by differences between manageable and non-manageable angles. However, regarding absolute RT values, those of younger children were generally longer, which could explain the more apparent RT differences between manageable and non-manageable angles in younger children. For this reason, we also performed the analysis using normalized RTs (see Data analyses) as described below.

### 4.4 Developmental changes in biomechanical constraints on response times

**Figure 7.**
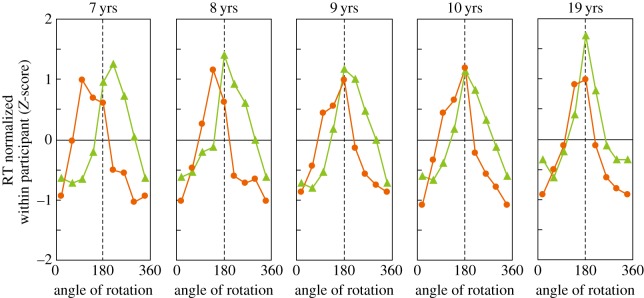
Mean normalized (*Z*-scored) RTs as a function of rotation angle in the picture plane. The same patterns of functions as figure 4 are observed with no absolute RT differences across ages.

To statistically examine whether biomechanical constraints on RTs are stronger for younger children, *Z*-scored RTs were calculated (figure 7). This normalization was conducted to detect pattern differences in RT–angle functions across ages after absolute age differences in RTs were removed. As shown in figure 7, the resulting *Z*-scored RTs more clearly captured developmental changes in biomechanical constraints.

**Figure 8.**
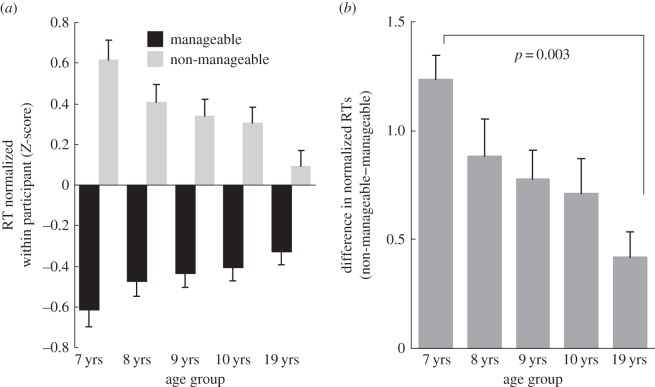
Mean normalized (*Z*-scored) RTs for manageable angles and non-manageable angles. (*a*) The mean normalized RT was longer for non-manageable angles than for manageable angles in each age group. (*b*) The difference in normalized RTs between manageable and non-manageable angles decreased with age.

Statistical analyses were conducted using the means of the normalized RTs for manageable (left hand: 45^°^–135^°^; right hand: 225^°^–315^°^) and non-manageable angles (left hand: 225^°^–315^°^; right hand: 45^°^–135^°^). Repeated measures ANOVA of manageability × laterality × group (2×2×5) showed significant main effects of manageability (*F*_1,62_=158.297, *p*<0.0001,*η*^2^=0.53) and laterality (*F*_1,62_=11.080, *p*=0.002,*η*^2^=0.03). As anticipated, the manageability × group interaction was significant (*F*_4,62_=3.904, *p*=0.007,*η*^2^=0.05), with larger manageability effects in younger children (figure 8*a*). Because no other effects were significant, including any interactions with laterality, the laterality factor collapsed in figure 8. The results indicate that participants responded to manageable angles faster (*Z*-scores were below the mean, i.e. zero) than to non-manageable angles (*Z*-scores are above mean). In addition, the level of difference between manageable angles and non-manageable angles changed across age groups.

The difference between the mean normalized RT for manageable angles and for non-manageable angles was calculated for each age group (figure 8*b*). One-way ANOVA (five groups) identified a significant main effect of group (*F*_4,62_=3.904, *p*=0.007,*η*^2^=0.2), with larger differences between manageable and unmanageable angles for younger groups. Subsequent paired comparisons (Bonferroni) revealed that the 7-year-olds showed a significantly larger difference than adults (*p*=0.003). The closer neighbouring pairs were not significantly different.

Taken together, the more asymmetric RT–angle functions in younger children (figure 7) and the greater RT differences between manageable angles and non-manageable angles (figure 8) among younger children clearly indicate that the biomechanical properties of the body more strongly constrain their bodily imagery.

## 5. Discussion

The purpose of this study was to elucidate the developmental trajectory of body-related imagery, with the hypothesis that younger children's use of body schema relies more heavily on information related to action execution in the hand MR task than older children. Specifically, we focused on biomechanical constraints on RTs for the task. We used a standard hand MR task in which rotated hand pictures were presented one at a time so that each one was compared with each participant's body schema. To ensure that motor imagery was evoked, we introduced depth variation by presenting the back, thumb-side and palm of each hand. The results supported the hypothesis in several ways.

### 5.1 Stronger biomechanical effects and execution-related properties in younger children

First, as revealed in the proportion of participants who passed, moved or failed, the 6-year-olds had difficulty in following the instruction not to move their hands during the task. This finding suggests that it is difficult for 6-year-olds to manipulate body schema without the assistance of action execution. Second, error rates and RTs indicated stronger biomechanical constraints on body-related imagery in younger children (especially the 7-year-olds) compared with older children and adults. That is, both error rates and RTs indicated that the older children and adults showed smaller differences between manageable and non-manageable angles, suggesting a developmental weakening with age of reliance on properties of action execution. These results suggest that there is a transitional period (around 7–8 years) during which children shift the way by which they manipulate body schema from action execution to motor imagery. In addition, older children and adults seemed to manipulate body-related imagery in a more visual way compared with young children, suggesting their ability to use multiple sources of information as visuomotor imagery. Such a visuomotor nature of older participants' manipulation of body schema may represent maturation of the dorsal premotor cortex, which receives multimodal inputs, including visual, somatosensory and attentional signals [30,43] and serves for motor planning [44,45].

The present results are inconsistent, however, with those of Krüger & Krist [35], who did not find developmental weakening of biomechanical constraints. The disparity could be attributable to differences in experimental procedures, including the nature of the task: our task was to compare a stimulus with one's own body schema, whereas they used a matching-to-sample task within multiple stimuli. Another difference was the number of rotation axes: ours had two axes by introducing the depth variation (back, thumb-side, palm) in addition to the picture plane rotation, in contrast to only the latter in their study. According to ter Horst *et al*. [41], when the rotation axis is limited to one dimension, motor imagery is difficult to evoke. In addition, data analyses also differed between their study and ours: they used all RTs, but we used RTs only for correct responses. One or more of these differences in experimental procedure and data analyses may be responsible for the inconsistency between the two investigations. Another possibility is that their youngest participants (5 years old) might have moved their own hands during the task to some extent. If so, the imitative movements will facilitate the task and RTs would not accurately represent ‘MR’ in the youngest group. This possibility is plausible based on the fact that their 5-year-olds were faster than the 7-year-olds in a non-manageable angle. Similarly, our 6-year-olds who moved their hands showed somewhat weakened biomechanical constraints on RTs compared with the 7-year-olds who performed the task without moving their hands (this was more pronounced in the analysis by age grouping in electronic supplementary material than that by school grade grouping).

### 5.2 Awkward mental imagery in young children

Caeyenberghs *et al*. [34] examined the relationship between motor control ability and motor imagery in children (ages 7–8, 9–10 and 11–12 years) and found that the correlation between RTs for the hand MR task and motor skill scores was significant only for the oldest group. They argued that younger children have difficulty generating forward models of movement [46]. These results appear to be consistent with ours in that our younger participants were awkward in mental manipulation of body schema and required overt action assistance. Thus, younger children could not manipulate motor imagery as accurately as they could for overt action. As has been demonstrated in a non-MR study comparing the accuracy of imagined and executed actions [47], accuracy of imagined actions increases with age. The increasing correlation between motor imagery and motor execution is therefore likely to be a consequence of increasing imagery accuracy. The same relation could also be true for similar studies reporting correlations between imagined and executed actions for a pointing task based on a Fitts' law paradigm [18,48] .

### 5.3 Possible neural bases for action simulation

Our ‘primary area hypothesis’ started from differential maturation of various brain regions, that is, the dorsal premotor area critical to the hand MR task matures late (by adolescence, based on [26]), while the primary motor and somatosensory areas mature earliest (by 5 years). Based on our hypothesis, the stronger biomechanical constraints on young children were predicted as a manifestation of compensation by the early matured primary motor/somatosensory areas. The results supported the prediction, revealing stronger motor aspects in younger children. The results are also consistent with those by Funk *et al.* [33], that is, somatosensory information affects young children more than adults during the hand MR task. Our results, combined with those by Funk and colleagues, suggest a stronger motor aspect in young children's bodily imagery, and therefore a heavier involvement of brain regions for motor execution in young children, such as the primary motor/somatosensory areas [49]. In fact, while the dorsal premotor cortex is the most important neural correlate of visuomotor imagery in adults [12,20–24], the primary motor/somatosensory areas have been also implicated in the hand MR task in a few adult studies [23,25], similar to studies on motor imagery using other tasks [50–52]. Therefore, adults may also use the primary motor/somatosensory areas to a lesser extent for the hand MR task. The present results suggest that young children heavily recruit the primary areas which have a secondary role for adults.

### 5.4 Contrasts to adolescence and atypical development

In a recent study, developmental changes in biomechanical constraints in the hand MR task were examined across adolescence [53]. Those authors found weaker biomechanical constraints in younger adolescents when RTs were compared between 90^°^ and 270^°^. Although this result appears to be inconsistent with ours, the age ranges of the two studies do not overlap (they compared three age groups: 11–12, 14–15, 17–18 years old, while ours used 6-, 7-, 8-, 9-, 10-year-olds and adults). As older children acquire the ability for visual representation [31], there may be a period in which adolescents focus on visual imagery, while adults are more flexible and use the most efficient strategy such as visuomotor imagery. The experimental settings also differed in some respects, such as number of stimuli (they used two, a palm and back, and we used a palm, back and thumb-side) and rotation angles (they used four orientations based on a 90^°^-step rotation from upright, and we used eight orientations). Some of these differences may have led to the divergent outcomes.

Some populations with physical clumsiness as a result of developmental conditions do not exhibit biomechanical constraints on the hand MR task (e.g. in Asperger's syndrome [27]; in developmental motor coordination disorder [28,29]). Because many brain systems are affected in Asperger's syndrome and developmental motor coordination disorder [54,55], brain systems necessary for motor imagery are perhaps damaged or immature, which may lead to an adaptive process to develop an alternative strategy, such as purely visual imagery. The disappearance of biomechanical constraints in these populations suggests their difficulties in using body schema and a developmental strategy to compensate for it by alternative representation systems. These studies have tested participants in late childhood (age 9 or 10 years in [28,29]) or adolescence (13 years in [27]). It will be of interest to investigate how these populations perform the hand MR task in early childhood.

### 5.5 Developmental trajectory of manipulation of body schema

From the present results, the developmental trajectory of manipulation of body schema may be described as follows. First, as shown in our 6-year-olds, young children have difficulty in purely mental manipulation, thus need assistance of action execution to activate and manipulate body schema. At around 7–8 years, young children become capable of mental manipulation of body schema, but the mental process is strongly constrained by action execution properties, as indicated by the strong biomechanical constraints. Third, starting around 9 years, older children are more adult-like, with weakened but persistent biomechanical constraints on mental manipulation of body schema, suggesting use of visuomotor imagery, that is, action simulation which is more coordinated with visual transformation.

These three stages may be respectively characterized by action execution, motor imagery and visuomotor imagery. In this developmental trajectory, motor imagery in young children may have a role as a transitional period between action execution and purely mental manipulation of body schema as visuomotor imagery.

The transition from dominance of motor imagery to visuomotor imagery may be promoted by everyday visuomotor experiences and subsequent improvements in visuomotor control: a notable improvement of fine motor control has been found between 7–8 years and 9–10 years [34]. In addition, a general tendency of sensory development may also affect this transition, that is, tactile development precedes visual development, and multisensory development follows unimodal development, as revealed in animal neural responsiveness [56,57]. Finally, the transition from motor to visuomotor imagery may be associated with some other cognitive abilities such as working memory and executive functions, which improve substantially between 8 and 10 years [58,59].

## 6. Conclusion

In summary, this study revealed that younger children's use of body schema is more strongly constrained by properties of action execution, which was confirmed in two respects. First, the 6-year-olds strongly relied on overt action in the hand MR task. Second, once overt action became unnecessary, biomechanical constraints affected younger children's mental manipulation of body schema more strongly, and these constraints weakened later in childhood. These results indicate a transitional period (around 7 and 8 years of age) during which children shift from action execution to imagery for manipulating body schema. The results suggest that mental imagery of hands has a motor aspect more strongly in the transitional period than later in childhood and adulthood.

To conclude, we argue that young children strongly rely on properties accompanying action execution in manipulation of body schema. Older children and adults presumably manipulate body schema in an efficient way as visuomotor imagery. When body-related imagery begins to function in development, young children may mentally transform it as motor imagery with very weak visual properties. This study is the first to delineate the developmental transition in manipulating body schema in childhood, with three stages of action execution, motor imagery and visuomotor imagery. Because body schema would be fundamental for action-perception links and embodied cognition, the present findings significantly advance our understanding of cognitive development and provide suggestions on how to teach young children. We tentatively propose the primary area hypothesis to account for younger children's greater reliance on properties of action execution, but of course, testing of this hypothesis is an aim for future investigations.

## Supplementary Material

Suppementary_MR.PDF This file includes a set of results of alternative analyses (Supplementary material 1 to 3) as well as the data supporting this paper, that is, error rate and mean response time in each condition for each participant (Supplementary material 4 and 5)

## Data accessibility

The datasets supporting this article have been uploaded as part of the electronic supplementary material.
