# Supplementary material for: Strong biomechanical constraints on young children's mental imagery of hands
Source: R Soc Open Sci. 2014 Dec 10;1(4):140118. doi: 10.1098/rsos.140118 (PMC4448770; doi:10.1098/rsos.140118)
Supplement: Suppementary_MR.PDF This file includes a set of results of alternative analyses (Supplementary material 1 to 3) as well as the data supporting this paper, that is, error rate and mean response time in each condition for each participant (Supplementary material 4 and 5) [file rsos140118supp1.pdf]

Supplementary Material 1

Participants categorized by chronological age

Table 1S: Chronological age groups (n = 99)

Group	n	Mean age	male/female
6 years	15	6.70	10/5
7 years	14	7.54	4/10
8 years	21	8.46	17/4
9 years	14	9.54	7/7
10−11 years	20	10.68	15/5
19−20 years	15	19.62	11/4

Supplementary material 2

Tests for task execution of chronological age groups

We similarly counted the number of participants who performed the task significantly above chance-level accuracy without moving their own hands (‘pass’) in each age group. Participants who performed the task above chance but moved their own hands (‘move’) and those who performed below chance (‘fail’) were differently categorized. Distributions of participants were compared with a chi-square test (3 response categories × 6 groups).

As shown in Figure 1S, more children tended to yield failure in task execution or move their hands in younger age groups. In particular, 6-year-olds showed highly degraded performance, as observed similarly in the main analysis for school grade groups. The overall chi-square test showed a significant group effect ($\chi^2_{(10)} = 72.722$, $p < 0.0001$). Here, 6-year-old children were also considerably different in task performance from any other age group (all of $\chi^2_{(2)} > 15.443$, all p values < 0.0004). That is, it was confirmed in the analysis for chronological age groups that the standard hand MR task is especially difficult for 6-year-olds, as was shown in the main analysis for school year groups.

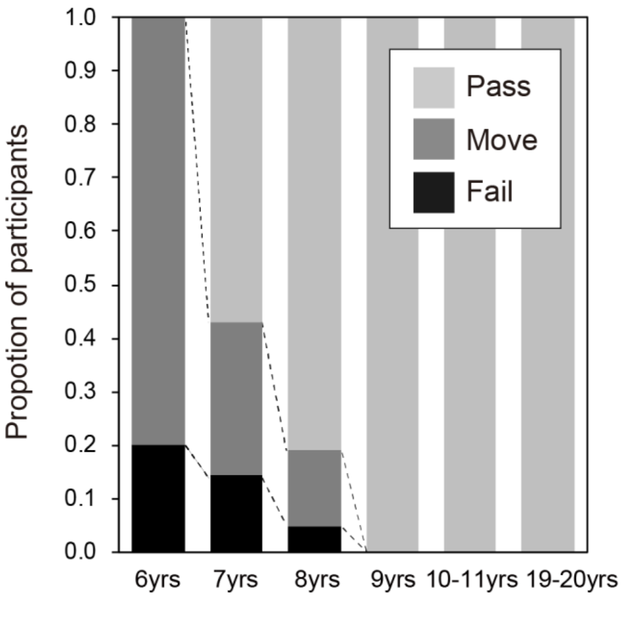

Figure 1S

Supplementary material 3

Function of RT by angles in each of chronological age group

As in the main analysis for school grade groups, we plotted pattern differences in the RT–angle (Figure 2S) and error rate-angle (Figure 3S) relationships to inspect developmental changes in biomechanical constraints on the hand MR. The biomechanical constraints on the peak angle of the RT-angle function (separation between peaks for the right and left hands) were the most salient in the 7-year-olds, and a developmental weakening followed. In this age grouping, the 6-year-olds showed a weakened biomechanical effect compared with the 7-year-olds, perhaps due to all of the 6-year-olds moved their hands during the task while the data for the 7-year-olds in Figure 2S are only for those who passed the criteria without moving hands. Because only a small number of the 7-year-olds (n = 4) reached the criteria for analyzing RTs, we concentrated on error rates for statistical analyses.

For error rate analyses (n = 99, all the participants included), a chi-square test was performed similarly with the main analysis. Individual correct and incorrect responses were counted for manageable and non-manageable angles across both hands. The manageable/non-manageable ratio of (1 – error rate) was also calculated after logarithmical transformation of percent correct for each individual. Group differences in this ratio were examined using the Kruskal–Wallis test. Following paired comparisons between groups were conducted with the Mann–Whitney test.

The biomechanical constraints instantiated by asymmetry of error rate functions were also salient for younger age groups in Figure 3S. In the analyses for error rates, all age groups made more errors in non-manageable angles (6-years: $\chi^2_{(1)} = 197.998$, $p < 0.0001$; 7-years: $\chi^2_{(1)} = 138.525$, $p < 0.0001$; 8-years: $\chi^2_{(1)} = 141.704$, $p < 0.0001$; 9-years: $\chi^2_{(1)} = 25.810$, $p < 0.0001$; 10-11-years: $\chi^2_{(1)} = 65.581$, $p < 0.0001$; 19-years: $\chi^2_{(1)} = 8.176$, $p = 0.004$) (Figure 4AS). In paired comparisons of manageable/non-manageable ratios among age groups, 6-, 7-, and 8-year-old children yielded higher ratios (that is, higher correct response to manageable angles) than older children and adults (6-years: all of *Us* < 68.5, all of *p-values* < 0.006; 7-years: all of *Us* < 60.5, all of *p-values* < 0.005; 8-years: all of *Us* < 123.0, all of *p-values* < 0.023) (Figure 4BS). These results suggest that the biomechanical constraints to the hand MR are stronger until around 8 years of age.

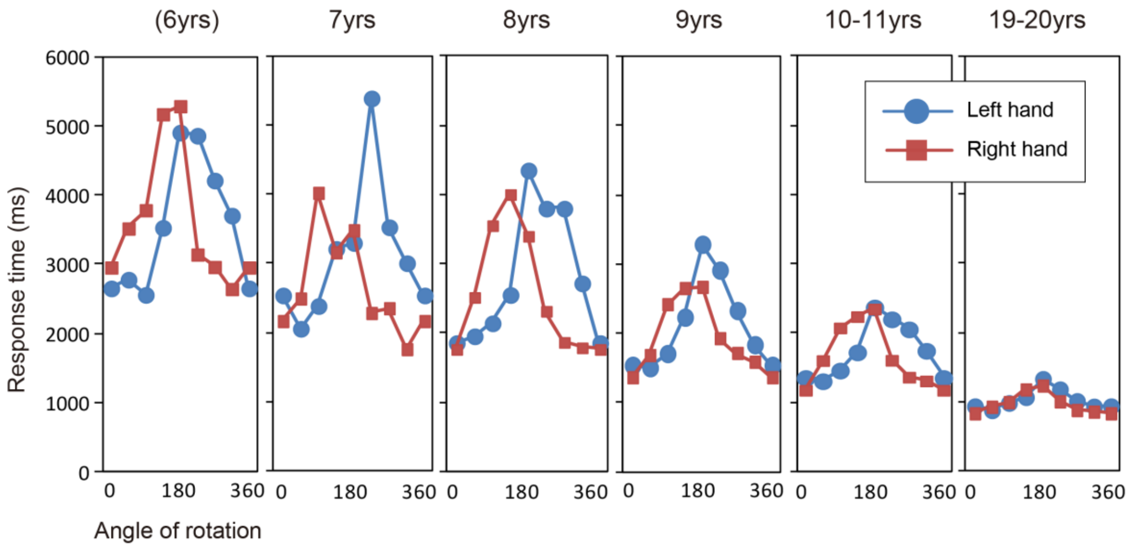

Figure 2S

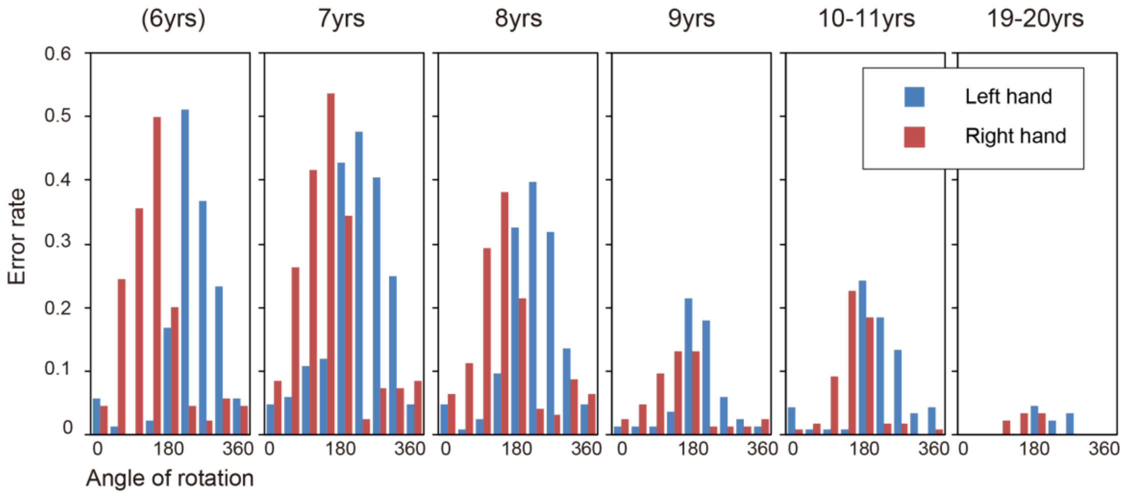

Figure 3S

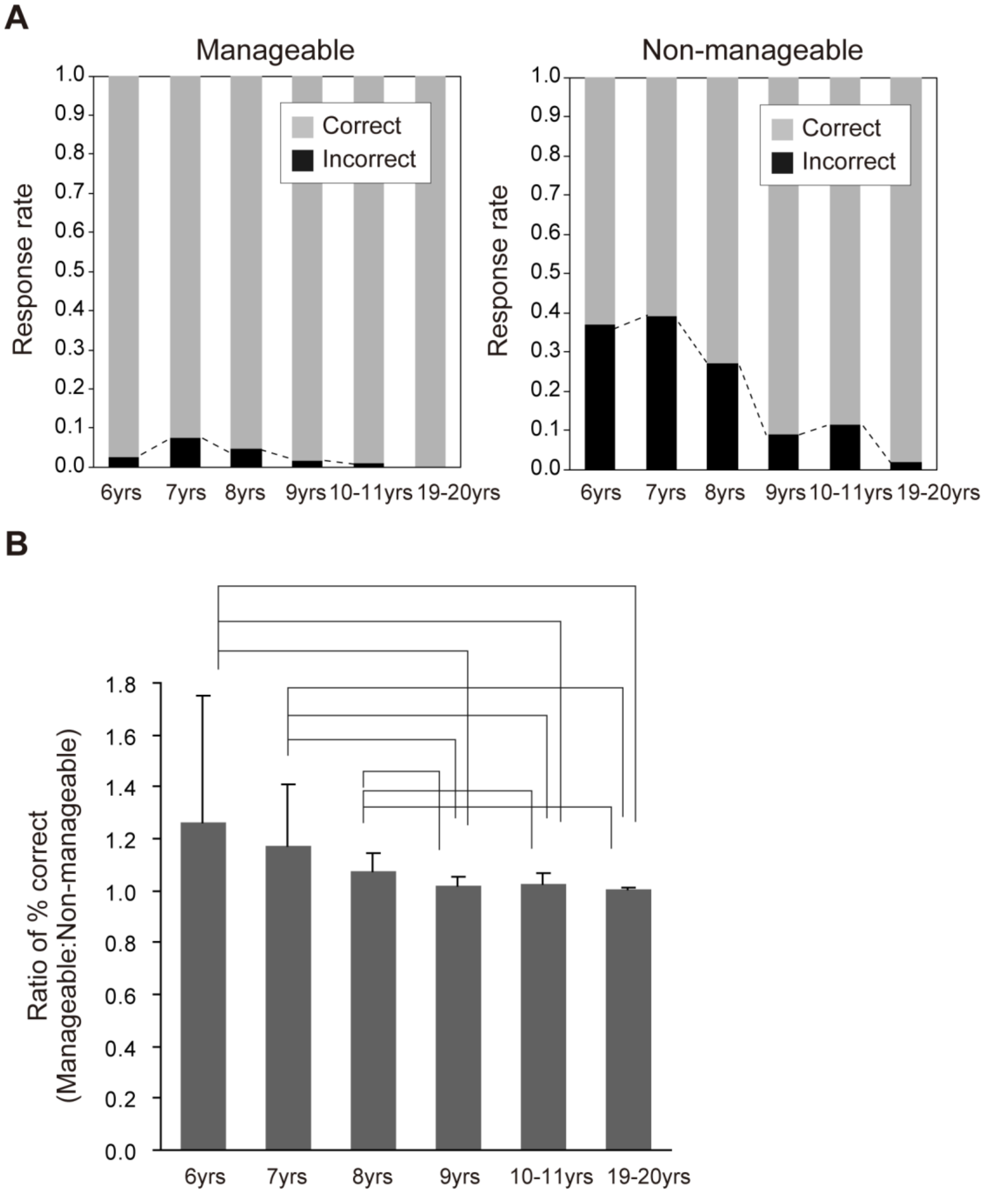
A
Manageable
Non-manageable
Correct
Incorrect
Response rate
1.0
0.9
0.8
0.7
0.6
0.5
0.4
0.3
0.2
0.1
0.0
6yrs
7yrs
8yrs
9yrs
10-11yrs
19-20yrs
B
Ratio of % correct
(Manageable:Non-manageable)
1.8
1.6
1.4
1.2
1.0
0.8
0.6
0.4
0.2
0.0

Figure 4S

Supplementary Material 4

Error ratios for mental rotation of left and right hands in 8 angles in Experiment 2 (n=99).

Group	Samples for error ratio alanlysis (n=99)	Sex	Age	Left hand (angle)								Right hand (angle)							Manageabe	non–manageable
				0	45	90	135	180	225	270	315	45	90	135	180	225	270	315		
1	1	m	6.41	0.00	0.00	0.00	0.00	0.00	0.67	0.33	0.17	0.33	0.67	0.50	0.17	0.00	0.00	0.00	0.00	0.44
1	2	m	6.50	0.00	0.00	0.00	0.00	0.00	0.50	0.17	0.00	0.00	0.17	0.33	0.17	0.00	0.00	0.17	0.03	0.19
1	3	f	6.59	0.00	0.00	0.00	0.00	0.00	0.67	0.67	0.17	0.17	0.17	0.50	0.33	0.00	0.00	0.33	0.06	0.39
1	4	m	6.59	0.33	0.17	0.00	0.00	0.17	0.83	0.50	0.17	0.50	0.33	0.67	0.33	0.00	0.00	0.17	0.06	0.50
1	5	m	6.63	0.00	0.00	0.00	0.17	0.33	0.33	0.50	0.50	0.00	0.17	0.67	0.33	0.00	0.17	0.00	0.06	0.36
1	6	m	6.66	0.00	0.00	0.00	0.00	0.00	0.67	0.33	0.33	0.33	0.67	0.50	0.17	0.00	0.00	0.00	0.00	0.47
1	7	m	6.68	0.17	0.00	0.00	0.00	0.00	0.50	0.17	0.00	0.00	0.50	0.33	0.00	0.33	0.00	0.00	0.06	0.25
1	8	m	6.75	0.00	0.00	0.00	0.00	0.00	0.00	0.00	0.00	0.00	0.00	0.17	0.00	0.00	0.00	0.17	0.03	0.03
1	9	f	6.78	0.00	0.00	0.00	0.00	0.33	0.00	0.00	0.00	0.00	0.00	0.00	0.00	0.00	0.00	0.00	0.00	0.00
1	10	f	6.84	0.00	0.00	0.00	0.00	0.33	0.67	0.17	0.17	0.00	0.00	0.67	0.50	0.00	0.00	0.00	0.00	0.28
1	11	m	6.89	0.00	0.00	0.00	0.00	0.00	0.00	0.00	0.00	0.00	0.00	0.00	0.00	0.00	0.00	0.00	0.00	0.00
1	12	m	6.99	0.00	0.00	0.00	0.17	0.17	0.50	0.17	0.17	0.00	0.33	0.17	0.17	0.33	0.17	0.00	0.11	0.22
1	13	f	7.12	0.00	0.00	0.00	0.00	0.50	0.83	0.50	0.33	0.17	0.17	0.50	0.00	0.00	0.00	0.17	0.03	0.42
1	14	f	7.18	0.67	0.33	0.67	0.50	0.83	0.83	0.83	0.67	0.83	0.83	0.67	0.33	0.00	0.17	0.00	0.28	0.78
1	15	m	7.24	0.00	0.00	0.00	0.00	0.00	0.00	0.00	0.33	0.00	0.00	0.33	0.00	0.00	0.00	0.00	0.00	0.11
1	16	f	7.28	0.00	0.00	0.00	0.00	0.00	0.00	0.00	0.00	0.17	0.00	0.17	0.00	0.00	0.00	0.00	0.00	0.06
1	17	f	6.42	0.17	0.00	0.00	0.00	0.50	1.00	0.83	0.67	1.00	1.00	1.00	0.67	0.00	0.00	0.00	0.00	0.92
1	18	m	6.75	0.17	0.00	0.00	0.00	0.17	1.00	1.00	1.00	1.00	0.67	1.00	0.00	0.00	0.00	0.00	0.00	0.94
1	19	f	6.97	0.00	0.00	0.00	0.00	0.50	0.33	0.67	0.17	0.33	0.67	1.00	0.17	0.00	0.00	0.00	0.00	0.53
1	20	f	7.16	0.00	0.00	0.17	0.17	0.50	0.83	0.50	0.67	0.33	0.67	0.83	1.00	0.00	0.00	0.17	0.08	0.64
2	21	f	7.59	0.00	0.00	0.17	0.17	0.33	0.50	0.33	0.00	0.17	0.50	0.17	0.33	0.00	0.00	0.00	0.06	0.28
2	22	f	7.64	0.00	0.17	0.00	0.00	0.83	0.50	0.33	0.33	0.00	0.83	0.83	0.83	0.00	0.00	0.00	0.03	0.47
2	23	m	7.70	0.00	0.00	0.17	0.50	0.67	0.00	0.83	0.00	0.50	0.67	0.50	0.00	0.00	0.83	0.33	0.31	0.42
2	24	f	7.76	0.00	0.33	0.33	0.00	0.33	0.33	0.33	0.17	0.50	0.33	0.83	0.50	0.33	0.00	0.33	0.22	0.42
2	25	m	7.86	0.00	0.00	0.00	0.00	0.00	0.33	0.00	0.00	0.17	0.00	0.17	0.00	0.00	0.00	0.00	0.00	0.11
2	26	m	7.93	0.00	0.00	0.00	0.17	0.33	0.00	0.00	0.00	0.00	0.00	0.00	0.33	0.00	0.00	0.00	0.03	0.00
2	27	m	8.01	0.00	0.00	0.00	0.00	0.67	0.33	0.67	0.00	0.00	0.00	0.33	0.00	0.00	0.00	0.00	0.00	0.22
2	28	m	8.12	0.00	0.00	0.00	0.00	0.00	0.17	0.33	0.33	0.17	0.17	0.50	0.17	0.17	0.00	0.00	0.03	0.28
2	29	m	8.20	0.00	0.00	0.17	0.00	0.00	0.00	0.17	0.00	0.00	0.00	0.33	0.00	0.00	0.00	0.00	0.03	0.08
2	30	m	8.35	0.00	0.00	0.00	0.17	0.33	0.33	0.33	0.00	0.00	0.17	0.00	0.83	0.00	0.00	0.17	0.06	0.14
2	31	f	7.67	0.00	0.00	0.00	0.00	0.83	0.83	0.67	0.33	0.17	0.50	0.83	0.17	0.00	0.00	0.00	0.00	0.56
2	32	f	7.83	0.00	0.00	0.00	0.17	0.00	0.67	0.33	0.00	0.00	0.33	0.67	0.50	0.00	0.00	0.00	0.03	0.33
2	33	m	8.00	0.00	0.00	0.00	0.00	0.00	0.17	0.00	0.17	0.00	0.17	0.17	0.00	0.00	0.00	0.00	0.00	0.11
2	34	m	8.08	0.00	0.00	0.00	0.00	0.33	0.67	0.67	0.00	0.00	0.50	0.67	0.67	0.00	0.00	0.00	0.00	0.42
2	35	m	8.17	0.33	0.00	0.00	0.17	0.50	0.33	0.67	0.50	0.50	0.67	0.50	0.17	0.00	0.00	0.00	0.03	0.53
2	36	f	7.61	0.00	0.00	0.00	0.00	0.83	1.00	1.00	0.67	0.67	1.00	1.00	0.83	0.00	0.00	0.00	0.00	0.89
2	37	f	8.33	0.00	0.00	0.00	0.00	0.33	0.67	0.67	0.00	0.17	0.67	0.33	0.33	0.17	0.00	0.00	0.03	0.42
2	38	m	8.36	0.00	0.00	0.00	0.00	0.33	0.00	0.33	0.00	0.00	0.00	0.00	0.00	0.00	0.00	0.00	0.00	0.06
3	39	m	8.39	0.00	0.00	0.00	0.17	0.00	0.00	0.00	0.17	0.33	0.00	0.33	0.17	0.00	0.33	0.33	0.14	0.14
3	40	m	8.43	0.00	0.00	0.00	0.17	0.33	0.50	0.00	0.00	0.00	0.00	0.00	0.17	0.00	0.00	0.67	0.14	0.08
3	41	m	8.44	0.00	0.00	0.00	0.33	0.33	0.67	0.17	0.50	0.17	0.33	0.50	0.33	0.00	0.00	0.00	0.06	0.39
3	42	f	8.53	0.00	0.00	0.17	0.17	0.33	0.50	0.33	0.00	0.17	0.50	0.17	0.33	0.00	0.00	0.00	0.06	0.28
3	43	m	8.67	0.17	0.00	0.17	0.17	0.83	0.83	0.33	0.17	0.17	0.67	0.67	0.00	0.00	0.17	0.00	0.08	0.47
3	44	m	8.67	0.17	0.00	0.00	0.00	0.50	0.50	0.17	0.17	0.17	0.50	0.83	0.00	0.00	0.00	0.50	0.08	0.39
3	45	m	8.79	0.00	0.00	0.00	0.00	0.67	0.33	0.00	0.00	0.00	0.17	0.67	0.17	0.00	0.00	0.00	0.00	0.19
3	46	m	8.89	0.00	0.17	0.00	0.00	0.00	0.17	0.00	0.00	0.00	0.00	0.00	0.00	0.00	0.17	0.00	0.06	0.03
3	47	f	8.93	0.00	0.00	0.00	0.00	0.33	0.50	0.50	0.17	0.00	0.00	0.17	0.17	0.00	0.00	0.00	0.00	0.22
3	48	m	8.94	0.00	0.00	0.00	0.00	0.00	0.33	0.17	0.00	0.00	0.00	0.17	0.17	0.17	0.00	0.00	0.03	0.11
3	49	f	9.11	0.00	0.17	0.00	0.17	0.00	0.00	0.00	0.00	0.00	0.00	0.00	0.00	0.00	0.00	0.17	0.08	0.00
3	50	f	9.18	0.00	0.00	0.00	0.17	0.17	0.17	0.00	0.17	0.00	0.17	0.17	0.50	0.00	0.17	0.00	0.06	0.11
3	51	m	9.29	0.00	0.00	0.00	0.00	0.67	0.67	0.67	0.00	0.17	0.50	0.17	0.00	0.00	0.00	0.00	0.00	0.36
3	52	m	9.30	0.00	0.00	0.00	0.00	0.00	0.00	0.00	0.00	0.00	0.00	0.17	0.00	0.00	0.00	0.00	0.00	0.03
3	53	f	8.74	0.00	0.00	0.00	0.17	0.67	0.67	0.67	0.17	0.33	0.83	0.83	0.50	0.00	0.00	0.00	0.03	0.58
3	54	m	8.57	0.33	0.00	0.00	0.50	0.33	0.67	0.50	0.50	0.17	0.83	0.83	0.33	0.33	0.00	0.17	0.17	0.58
4	55	m	9.43	0.00	0.00	0.00	0.00	0.00	0.00	0.00	0.00	0.00	0.00	0.00	0.00	0.00	0.00	0.00	0.00	0.00
4	56	f	9.44	0.00	0.00	0.00	0.00	0.50	0.17	0.00	0.00	0.00	0.00	0.17	0.17	0.00	0.00	0.00	0.00	0.06
4	57	f	9.50	0.00	0.00	0.00	0.00	0.67	0.17	0.00	0.00	0.17	0.17	0.17	0.33	0.17	0.00	0.00	0.03	0.11
4	58	m	9.57	0.00	0.00	0.00	0.00	0.00	0.00	0.00	0.00	0.00	0.00	0.00	0.00	0.00	0.00	0.00	0.00	0.00
4	59	f	9.59	0.00	0.00	0.00	0.00	0.00	0.33	0.00	0.00	0.00	0.00	0.00	0.00	0.00	0.00	0.00	0.00	0.06
4	60	m	9.69	0.00	0.00	0.00	0.00	0.33	0.50	0.17	0.17	0.33	0.33	0.50	0.50	0.00	0.00	0.00	0.00	0.33
4	61	f	9.76	0.00	0.00	0.00	0.00	0.17	0.17	0.00	0.00	0.00	0.00	0.17	0.00	0.00	0.00	0.00	0.00	0.06
4	62	f	9.86	0.17	0.00	0.17	0.17	0.50	0.33	0.00	0.00	0.00	0.17	0.33	0.33	0.00	0.00	0.00	0.06	0.14
4	63	m	9.88	0.00	0.00	0.00	0.00	0.00	0.00	0.00	0.00	0.00	0.00	0.00	0.00	0.00	0.00	0.00	0.00	0.00
4	64	m	9.99	0.00	0.00	0.00	0.00	0.00	0.00	0.00	0.00	0.00	0.00	0.00	0.00	0.00	0.00	0.00	0.00	0.00
4	65	m	10.00	0.17	0.00	0.00	0.00	0.17	0.33	0.00	0.00	0.00	0.00	0.50	0.00	0.00	0.00	0.00	0.00	0.14
4	66	m	10.14	0.00	0.00	0.00	0.00	0.50	0.33	0.17	0.00	0.00	0.00	0.33	0.17	0.00	0.00	0.00	0.00	0.14
4	67	m	10.14	0.00	0.00	0.00	0.00	0.00	0.00	0.00	0.00	0.00	0.00	0.00	0.00	0.00	0.00	0.00	0.00	0.00
4	68	m	10.28	0.00	0.00	0.00	0.00	0.33	0.33	0.33	0.00	0.17	0.17	0.33	0.00	0.00	0.00	0.00	0.00	0.22
4	69	f	10.29	0.00	0.00	0.00	0.00	0.33	0.33	0.00	0.00	0.00	0.17	0.50	0.67	0.00	0.00	0.00	0.00	0.17
5	70	f	10.38	0.00	0.17	0.17	0.00	0.33	0.00	0.17	0.00	0.00	0.00	0.00	0.00	0.00	0.17	0.00	0.08	0.03
5	71	m	10.44	0.17	0.00	0.00	0.00	0.00	0.00	0.00	0.00	0.00	0.00	0.00	0.00	0.17	0.00	0.00	0.03	0.00
5	72	m	10.52	0.17	0.00	0.00	0.00	0.50	0.50	0.67	0.17	0.00	0.50	0.67	0.17	0.00	0.00	0.00	0.00	0.42
5	73	m	10.53	0.00	0.00	0.00	0.00	0.00	0.00	0.00	0.17	0.00	0.00	0.00	0.67	0.17	0.00	0.00	0.03	0.03
5	74	m	10.88	0.33	0.00	0.00	0.00	0.00	0.17	0.33	0.33	0.00	0.17	0.00	0.17	0.00	0.00	0.00	0.00	0.17
5	75	m	10.90	0.00	0.00	0.00	0.00	0.33	0.00	0.00	0.00	0.00	0.00	0.00	0.17	0.00	0.00	0.00	0.00	0.00
5	76	m	10.90	0.00	0.00	0.00	0.00	0.67	0.50	0.17	0.00	0.00	0.50	0.67	0.83	0.00	0.00	0.00	0.00	0.31
5	77	m	11.04	0.00	0.00	0.00	0.00	0.00	0.17	0.00	0.00	0.00	0.00	0.00	0.00	0.00	0.00	0.00	0.00	0.03
5	78	m	11.09	0.00	0.00	0.00	0.17	0.67	0.67	0.50	0.00	0.17	0.17	0.50	0.00	0.00	0.17	0.00	0.06	0.33
5	79	f	11.26	0.00	0.00	0.00	0.00	0.33	0.17	0.00	0.00	0.00	0.00	0.33	0.33	0.00	0.00	0.00	0.00	0.08
5	80	m	11.27	0.00	0.00	0.00	0.00	0.00	0.00	0.00	0.00	0.00	0.00	0.00	0.00	0.00	0.00	0.00	0.00	0.00
5	81	f	11.37	0.00	0.00	0.00	0.00	0.00	0.00	0.00	0.00	0.00	0.00	0.00	0.00	0.00	0.00	0.00	0.00	0.00
5	82	f	10.75	0.00	0.00	0.00	0.00	0.67	0.17	0.33	0.00	0.00	0.17	0.50	0.50	0.00	0.00	0.00	0.00	0.19
5	83	m	11.17	0.00	0.00	0.00	0.00	0.00	0.00	0.00	0.00	0.00	0.00	0.00	0.00	0.00	0.00	0.00	0.00	0.00
5	84	m	10.33	0.00	0.00	0.00	0.00	0.00	0.00	0.00	0.00	0.00	0.00	0.17	0.00	0.00	0.00	0.00	0.00	0.03
UG	85	m	18.73	0.00	0.00	0.00	0.00	0.00	0.00	0.00	0.00	0.00	0.17	0.00	0.00	0.00	0.00	0.00	0.00	0.03
UG	86	m	18.96	0.00	0.00	0.00	0.00	0.17	0.00	0.00	0.00	0.00	0.00	0.00	0.00	0.00	0.00	0.00	0.00	0.00
UG	87	m	19.03	0.00	0.00	0.00	0.00	0.33	0.00	0.00	0.00	0.00	0.00	0.17	0.00	0.00	0.00	0.00	0.00	0.03
UG	88	f	19.21	0.00	0.00	0.00	0.00	0.00	0.00	0.00	0.00	0.00	0.00	0.00	0.00	0.00	0.00	0.00	0.00	0.00
UG	89	m	19.24	0.00	0.00	0.00	0.00	0.00	0.00	0.00	0.00	0.00	0.00	0.00	0.00	0.00	0.00	0.00	0.00	0.00
UG	90	m	19.26	0.00	0.00	0.00	0.00	0.00	0.00	0.00	0.00	0.00	0.00	0.00	0.00	0.00	0.00	0.00	0.00	0.00
UG	91	m	19.32	0.00	0.00	0.00	0.00	0.00	0.00	0.00	0.00	0.00	0.00	0.00	0.00	0.00	0.00	0.00	0.00	0.00
UG	92	m	19.50	0.00	0.00	0.00	0.00	0.00	0.00	0.33	0.00	0.00	0.00	0.00	0.00	0.00	0.00	0.00	0.00	0.06
UG	93	m	19.53	0.00	0.00	0.00	0.00	0.00	0.00	0.00	0.00	0.00	0.00	0.00	0.00	0.00	0.00	0.00	0.00	0.00
UG	94	m	19.53	0.00	0.00	0.00	0.00	0.00	0.00	0.00	0.00	0.00	0.17	0.00	0.00	0.00	0.00	0.00	0.00	0.03
UG	95	m	20.22	0.00	0.00	0.00	0.00	0.00	0.00	0.00	0.00	0.00	0.00	0.00	0.00	0.00	0.00	0.00	0.00	0.00
UG	96	m	20.49	0.00	0.00	0.00	0.00	0.00	0.00	0.00	0.00	0.00	0.00	0.17	0.00	0.00	0.00	0.00	0.00	0.03
UG	97	f	21.18	0.00	0.00	0.00	0.00	0.00	0.00	0.00	0.00	0.00	0.00	0.00	0.00	0.00	0.00	0.00	0.00	0.00
UG	98	f	19.75	0.00	0.00	0.00	0.00	0.17	0.33	0.17	0.00	0.00	0.00	0.00	0.33	0.00	0.00	0.00	0.00	0.08
UG	99	f	20.33	0.00	0.00	0.00	0.00	0.00	0.00	0.00	0.00	0.00	0.00	0.17	0.17	0.00	0.00	0.00	0.00	0.03

1: 1st grade; 2: 2nd grade; 3: 3rd grade; 4: 4th grade; 5: 5th grade; UG: under graduate; f: female; m: male;

Error ratios for manageable directions were calculated by averaging RTs for angles from 45° to 135° for the left hand and from 225° to 315° for the right hand.

Error ratios for non-manageable directions were calculated by averaging RTs for angles from 225° to 315° for the left hand and from 45° to 135° for the right hand.

Supplementary Material 5

Response time data for mental rotation of left and right hands in 8 angles in Experiment 2 (n=67).

Group	Samples for RT analysis (n=67)	Sex	Age	Left hand(angle)								Right hand(angle)								Left hand		Right hand	
				0	45	90	135	180	225	270	315	0	45	90	135	180	225	270	315	manage	nonmanage	manage	nonmanage
2	1	f	7.59	3.10	2.27	3.04	3.83	4.58	8.13	6.09	4.67	3.66	3.47	6.42	3.65	4.33	2.77	3.07	2.05	3.05	6.29	2.63	4.51
2	2	m	7.86	2.67	2.43	2.54	4.76	3.08	7.07	3.11	2.54	2.00	2.11	4.08	4.41	3.03	1.97	2.07	1.72	3.24	4.24	1.92	3.53
2	3	m	7.93	2.21	2.06	2.24	2.50	3.08	2.87	2.55	2.94	1.88	2.61	3.18	2.78	4.01	2.81	3.02	2.03	2.27	2.79	2.62	2.86
2	4	m	8.01	2.00	2.12	2.37	1.93	6.47	3.16	6.60	2.39	2.00	2.34	3.84	2.76	2.96	3.25	1.82	1.78	2.14	4.05	2.28	2.98
2	5	m	8.12	2.31	2.69	2.12	2.42	3.39	3.86	3.21	2.98	2.36	3.51	4.24	5.01	4.26	2.78	2.62	1.79	2.41	3.35	2.40	4.25
2	6	m	8.20	2.92	2.19	2.22	2.58	6.07	4.95	4.04	4.63	2.31	3.06	3.92	3.97	4.84	2.61	2.08	3.64	2.33	4.54	2.78	3.65
2	7	f	7.83	2.17	1.47	1.71	1.75	2.44	3.48	2.34	1.87	1.14	1.78	2.41	1.80	2.55	1.59	1.25	1.25	1.64	2.56	1.36	2.00
2	8	m	8.00	1.79	1.55	2.04	2.12	2.34	3.50	3.08	2.05	1.64	1.88	4.58	3.42	2.20	1.74	1.94	1.62	1.90	2.88	1.77	3.29
2	9	m	8.08	2.01	2.89	2.79	5.30	7.24	4.44	5.77	3.02	2.38	4.48	5.96	5.04	5.12	3.64	3.11	2.33	3.66	4.41	3.03	5.16
2	10	m	8.17	1.45	1.90	1.64	1.50	2.14	2.04	2.05	2.08	1.60	2.42	2.53	2.16	1.50	1.50	1.58	1.18	1.68	2.06	1.42	2.37
2	−	f	7.64	3.27	3.26	2.05	3.37	2.90	3.67	2.72	3.83	2.08	4.26	3.17	4.60	2.81	2.44	2.15	1.85	2.89	3.41	2.14	4.01
2	−	m	7.70	2.89	3.35	2.08	2.19	1.78	2.16	1.13	2.39	2.95	2.45	4.77	2.19	2.96	2.65	3.72	2.71	2.54	1.89	3.03	3.14
2	−	f	7.76	2.61	1.90	2.42	2.70	2.92	1.96	3.00	1.97	1.40	2.20	3.13	2.49	1.05	1.92	2.04	1.40	2.34	2.31	1.79	2.61
2	−	m	8.35	3.21	4.36	3.99	4.98	6.04	3.01	3.25	3.33	3.62	5.05	3.57	3.99	3.14	3.37	3.27	3.87	4.44	3.19	3.50	4.20
2	−	f	7.67	1.68	2.12	1.69	2.24	2.89	2.84	2.56	2.88	1.18	2.25	4.08	3.86	2.21	1.45	1.55	1.45	2.02	2.76	1.48	3.40
3	11	m	8.39	1.76	1.30	1.71	2.48	3.32	2.86	1.78	2.83	1.32	2.44	1.61	2.69	2.48	2.36	1.52	1.83	1.83	2.49	1.90	2.25
3	12	m	8.43	1.64	1.40	1.74	1.96	3.77	3.45	2.33	2.96	1.97	2.00	3.59	3.91	3.18	1.37	1.77	1.67	1.70	2.92	1.60	3.17
3	13	m	8.44	1.85	2.14	2.34	2.84	3.32	5.95	5.32	3.51	1.53	2.38	4.03	7.42	2.83	3.06	1.56	1.53	2.44	4.93	2.05	4.61
3	14	f	8.53	1.37	1.99	1.52	1.96	2.90	1.75	1.92	1.88	1.22	2.01	1.46	2.54	1.88	1.26	1.49	1.63	1.82	1.85	1.46	2.00
3	15	m	8.79	2.34	1.66	2.76	1.92	3.10	1.90	3.88	2.76	1.56	1.99	4.09	2.90	4.01	1.77	1.53	2.09	2.12	2.85	1.79	3.00
3	16	m	8.89	1.87	1.57	2.31	3.83	3.09	2.83	3.20	2.71	1.15	1.59	2.25	2.71	3.28	1.26	1.62	1.21	2.57	2.91	1.36	2.18
3	17	f	8.93	2.01	2.57	2.64	2.56	5.42	6.40	6.12	2.81	2.22	2.26	4.55	4.64	4.84	2.96	2.10	1.71	2.59	5.11	2.26	3.82
3	18	m	8.94	1.35	1.36	1.63	1.72	3.94	5.44	4.71	1.31	1.57	1.86	5.10	4.95	2.52	2.29	1.41	1.72	1.57	3.82	1.80	3.97
3	19	f	9.11	0.89	1.26	1.28	0.96	1.41	1.12	0.83	1.01	0.74	0.78	0.85	1.39	1.14	0.97	0.94	0.92	1.17	0.98	0.94	1.01
3	20	f	9.18	1.05	1.09	1.89	1.45	1.96	2.00	1.74	0.90	1.06	0.95	1.38	1.83	2.52	0.98	1.35	1.19	1.48	1.55	1.17	1.39
3	21	m	9.29	2.63	3.47	2.17	2.45	8.97	2.66	3.88	3.52	1.60	2.16	4.28	4.26	3.02	3.19	3.60	4.10	2.70	3.35	3.63	3.56
3	22	m	9.30	1.92	1.33	1.62	1.48	2.34	3.05	2.25	1.60	1.38	1.82	1.48	2.30	1.76	1.20	1.27	1.34	1.48	2.30	1.27	1.87
3	−	m	8.67	2.14	1.94	2.75	3.02	9.67	6.26	5.26	4.33	2.05	4.90	3.61	7.24	5.06	2.56	2.15	2.10	2.57	5.28	2.27	5.25
3	−	m	8.67	0.98	2.10	1.78	2.78	3.52	2.09	1.59	1.31	1.43	1.22	1.57	2.78	3.53	2.75	1.66	0.88	2.22	1.66	1.76	1.86
3	−	f	8.74	1.91	2.14	1.88	2.01	4.05	5.57	5.24	2.49	1.61	3.26	5.89	2.80	3.11	2.06	1.58	1.92	2.01	4.43	1.85	3.98
3	−	m	8.57	1.98	2.65	2.15	1.48	1.24	6.44	6.87	1.96	1.63	1.53	7.29	3.85	2.60	4.88	1.78	2.15	2.09	5.09	2.94	4.22
4	23	m	9.43	1.31	1.24	1.15	1.26	1.73	2.04	1.38	1.45	1.45	1.15	1.55	2.00	1.61	1.27	1.01	1.02	1.22	1.62	1.10	1.57
4	24	f	9.44	1.95	1.81	2.08	3.43	6.66	4.08	3.01	2.00	1.90	2.33	3.15	4.13	4.91	2.39	1.75	2.15	2.44	3.03	2.09	3.21
4	25	f	9.50	1.23	1.10	1.14	1.38	1.73	1.51	1.58	1.48	0.91	1.38	1.14	1.57	1.66	1.29	0.99	1.15	1.21	1.52	1.14	1.36
4	26	m	9.57	1.32	1.05	0.83	1.25	3.91	1.19	1.52	1.76	1.08	1.30	1.42	1.53	3.04	1.95	1.30	1.13	1.04	1.49	1.46	1.41
4	27	f	9.59	2.21	1.60	2.62	3.62	4.18	4.93	3.05	2.87	1.82	3.28	4.68	6.51	4.74	3.27	3.21	1.62	2.61	3.62	2.70	4.82
4	28	m	9.69	1.61	1.83	1.57	3.73	3.55	4.43	2.37	2.53	1.29	1.48	3.59	3.24	2.65	2.81	1.51	1.42	2.38	3.11	1.91	2.77
4	29	f	9.76	1.24	1.31	2.12	3.87	2.77	4.85	4.35	1.48	1.81	1.57	3.00	3.37	3.67	2.74	1.61	1.18	2.43	3.56	1.84	2.65
4	30	f	9.86	1.29	1.49	2.46	1.75	1.87	2.23	2.17	1.48	1.05	1.00	1.45	1.24	1.86	0.94	1.56	1.77	1.90	1.96	1.42	1.23
4	31	m	9.88	1.67	1.33	1.69	2.62	2.34	2.86	2.25	1.95	1.52	1.39	2.13	1.56	1.98	2.32	2.47	1.71	1.88	2.35	2.17	1.69
4	32	m	9.99	1.10	1.03	1.16	1.89	2.48	3.71	2.10	1.56	1.39	2.90	3.68	2.10	2.74	1.58	1.35	1.42	1.36	2.46	1.45	2.89
4	33	m	10.00	1.78	1.45	1.90	2.19	2.90	1.85	2.56	2.12	1.58	2.17	1.92	1.30	2.72	2.02	1.81	1.53	1.84	2.17	1.79	1.80
4	34	m	10.14	1.23	1.14	1.80	1.60	5.31	4.16	3.59	1.23	1.08	0.93	2.45	5.13	3.69	1.42	1.02	1.21	1.51	2.99	1.21	2.84
4	35	m	10.14	1.36	1.87	1.31	1.73	2.44	2.07	1.79	1.94	1.43	1.32	1.58	1.79	2.24	1.44	1.59	1.44	1.64	1.93	1.49	1.56
4	36	m	10.28	2.18	1.99	2.20	2.04	1.95	1.68	3.20	5.00	2.15	2.68	4.03	3.67	3.09	2.29	2.23	1.81	2.08	3.29	2.11	3.46
4	37	f	10.29	1.09	1.03	1.11	1.73	2.31	2.90	2.38	1.10	1.21	2.16	3.68	2.99	3.55	3.03	1.14	1.89	1.29	2.13	2.02	2.94
5	38	f	10.38	1.70	1.27	1.75	1.95	1.93	2.29	2.59	1.63	1.23	1.26	2.32	1.75	2.33	2.12	1.40	1.51	1.66	2.17	1.68	1.78
5	39	m	10.44	1.26	1.93	1.03	1.39	1.65	1.04	0.87	1.04	0.95	1.01	0.97	1.47	2.10	1.11	1.01	1.11	1.45	0.99	1.08	1.15
5	40	m	10.52	0.74	1.15	1.10	1.18	2.99	2.63	2.78	2.12	0.70	1.73	1.13	1.49	1.70	1.18	0.97	1.31	1.14	2.51	1.15	1.45
5	41	m	10.53	1.26	1.26	1.24	1.26	2.61	1.51	1.27	1.58	0.99	1.69	1.43	3.19	1.37	1.18	0.87	1.17	1.25	1.45	1.07	2.10
5	42	m	10.88	0.82	0.78	0.96	1.37	2.30	1.54	1.21	1.01	0.92	1.10	1.31	1.50	1.65	0.73	1.01	0.92	1.04	1.25	0.89	1.30
5	43	m	10.90	1.63	1.57	1.83	1.62	2.51	1.98	2.04	1.96	1.23	1.09	1.59	1.70	2.07	2.27	1.57	1.41	1.67	2.00	1.75	1.46
5	44	m	10.90	2.04	1.50	1.59	2.98	3.03	4.55	2.78	2.29	1.76	2.57	4.01	4.24	4.22	1.93	1.70	1.85	2.02	3.21	1.83	3.61
5	45	m	11.04	1.07	0.87	1.13	1.05	1.52	1.43	1.39	1.30	0.83	1.14	1.61	1.30	1.69	1.04	1.06	1.19	1.02	1.37	1.10	1.35
5	46	m	11.09	1.67	1.21	1.35	1.61	1.42	1.78	1.71	1.44	1.11	1.59	2.42	2.05	1.90	2.18	1.90	1.23	1.39	1.64	1.77	2.02
5	47	f	11.26	1.77	1.66	3.02	3.71	2.78	2.32	3.00	2.02	2.05	3.46	3.18	2.76	3.73	2.51	2.67	1.97	2.80	2.45	2.38	3.13
5	48	m	11.27	0.86	0.87	0.80	0.90	1.31	1.02	0.93	0.91	0.85	0.89	1.13	1.04	1.39	0.76	0.81	0.66	0.86	0.95	0.74	1.02
5	49	f	11.37	0.59	0.66	0.76	0.87	0.80	0.74	0.64	0.69	0.58	0.68	0.70	0.79	0.97	0.70	0.74	0.66	0.76	0.69	0.70	0.72
5	50	f	10.75	1.05	1.04	1.50	1.29	3.35	2.69	2.70	2.21	0.93	1.61	3.24	2.89	2.60	1.02	1.07	1.21	1.28	2.54	1.10	2.58
5	51	m	11.17	1.08	0.98	0.97	1.19	1.51	2.63	1.48	1.24	0.92	1.10	1.28	1.80	1.88	1.07	0.98	0.83	1.04	1.78	0.96	1.40
5	52	m	10.33	1.99	1.99	1.95	2.90	2.90	3.31	2.27	2.20	1.10	1.99	1.64	2.06	2.20	2.28	1.83	1.40	2.28	2.59	1.84	1.90
UG	53	m	18.73	0.90	0.93	1.27	1.01	1.39	1.07	0.90	1.00	1.02	1.06	0.97	1.31	1.54	0.97	0.96	0.88	1.07	0.99	0.94	1.11
UG	54	m	18.96	0.81	0.90	0.86	0.96	1.23	1.09	0.95	0.97	0.86	0.71	0.82	0.99	1.27	0.97	0.81	0.83	0.91	1.00	0.87	0.84
UG	55	m	19.03	0.92	0.81	0.81	0.86	1.12	1.05	0.89	0.95	0.74	0.71	0.97	1.00	0.90	0.84	0.80	0.77	0.83	0.96	0.80	0.89
UG	56	f	19.21	1.27	1.23	1.37	1.53	1.87	1.24	1.25	1.18	1.04	1.35	1.21	1.20	1.70	1.51	1.16	1.15	1.37	1.22	1.28	1.25
UG	57	m	19.24	1.18	1.26	1.07	1.36	1.34	1.34	1.20	1.11	1.15	1.14	1.02	1.38	1.22	1.24	1.10	1.04	1.23	1.22	1.13	1.18
UG	58	m	19.26	1.58	1.13	1.60	1.19	1.63	1.89	1.43	1.05	1.18	1.56	1.38	1.15	1.46	1.55	1.36	1.61	1.31	1.46	1.51	1.36
UG	59	m	19.32	0.89	0.80	0.85	0.86	1.42	1.74	1.18	0.94	0.90	0.93	1.23	1.87	1.79	1.09	0.92	0.84	0.84	1.29	0.95	1.34
UG	60	m	19.50	0.75	0.79	0.82	1.04	1.06	0.97	0.44	0.83	0.62	0.80	0.95	0.96	1.05	0.83	0.72	0.74	0.88	0.75	0.76	0.90
UG	61	m	19.53	0.59	0.53	0.54	0.62	0.84	0.65	0.63	0.61	0.52	0.59	0.59	0.74	0.72	0.60	0.65	0.56	0.56	0.63	0.60	0.64
UG	62	m	19.53	0.79	0.91	0.83	1.44	1.30	1.21	0.90	0.91	0.68	0.93	1.08	1.55	1.08	1.16	0.90	0.69	1.06	1.01	0.92	1.19
UG	63	m	20.22	0.62	0.58	0.62	0.74	0.73	0.66	0.57	0.58	0.53	0.52	0.60	0.70	0.60	0.56	0.59	0.53	0.65	0.60	0.56	0.61
UG	64	m	20.49	0.88	0.66	0.73	0.87	1.41	0.91	0.82	0.81	0.74	0.85	0.81	1.09	1.76	0.94	0.70	0.82	0.75	0.85	0.82	0.92
UG	65	f	21.18	0.84	0.74	0.98	0.92	1.42	1.18	0.98	0.93	0.80	0.98	0.99	1.28	0.96	0.89	0.82	0.93	0.88	1.03	0.88	1.08
UG	66	f	19.75	1.18	1.08	1.59	1.35	1.89	1.73	1.84	1.09	1.08	1.00	1.33	1.51	1.42	1.20	0.99	0.84	1.34	1.55	1.01	1.28
UG	67	f	20.33	0.88	0.84	0.85	1.26	1.36	1.01	1.22	1.05	0.68	0.83	1.00	0.98	1.09	0.77	0.76	0.67	0.99	1.09	0.73	0.94

2: 2nd grade; 3: 3rd grade; 4: 4th grade; 5: 5th grade; UG: under graduate; f: female; m: male;

RTs (sec) for manageable directions were calculated by averaging RTs for angles from 45° to 135° for the left hand or from 225° to 315° for the right hand.

RTs (sec) for non-manageable directions were calculated by averaging RTs for angles from 225° to 315° for the left hand or from 45° to 135° for the right hand.
